# Effect of vaccination against foot-and-mouth disease on milk yield in dairy cows

**DOI:** 10.1016/j.vas.2026.100683

**Published:** 2026-05-09

**Authors:** C. García-Pintos, A. Menchaca

**Affiliations:** a Fundación IRAUy, Instituto de Reproducción Animal Uruguay, Camino Cruz del Sur 2250, 11500 Montevideo, Uruguay; b Plataforma de Investigación en Salud Animal, Instituto Nacional de Investigación Agropecuaria (INIA), Estación Experimental La Estanzuela, Ruta 50, km 11, 70000 Colonia, Uruguay

**Keywords:** Holstein, Dairy cows, FMD vaccination, Milk yield, Hyperthermia

## Abstract

This study aimed to assess the impact of foot-and-mouth disease (FMD) vaccination on milk production in dairy cows. In Experiment 1, 593 lactating Holstein cows (37.2 ± 0.3 kg/day), housed in a free-stall system, were randomly assigned to receive (*n* = 268) or not receive (*n* = 325) a dose of the FMD vaccine on Day 0 of the experiment. Milk yield was recorded from Day −3 to Day 9, and body temperature was measured from Day 0 to Day 3. Among cows producing ≥ 37.2 kg/day, vaccinated animals had a significantly lower milk yield than unvaccinated controls from Days 1 to 5 post-vaccination (40.7 ± 0.3 kg/day *vs.* 42.9 ± 0.3 kg/day; *P* < 0.05). In cows producing < 37.2 kg/day, milk yield was lower in vaccinated animals on Days 1 and 2 (31.0 ± 0.3 kg/day *vs*. 32.9 ± 0.3 kg/d; *P* < 0.05), with no differences detected thereafter. In a subset of cows from Experiment 1 (*n* = 96), vaccinated cows had higher body temperature on Day 1 (39.1 ± 0.1 °C *vs.* 38.2 ± 0.1 °C; *P* < 0.05). In Experiment 2, 146 lactating Holstein cows (20.3 ± 0.3 kg/day), managed on a pasture-based system, were randomly assigned to receive (*n* = 78) or not receive (*n* = 68) a dose of the FMD vaccine on Day 0. Milk yield was measured on Days 0 and 3, and milk composition, somatic cell count, and milk urea nitrogen were analyzed on Day 3. No significant differences were observed between experimental groups for any of these variables. In conclusion, FMD vaccination caused only a transient milk yield reduction in high-producing cows (∼40 kg/day, free-stall system), but no significant effect was seen in lower-producing cows (∼20 kg/day, pasture-based system).

## Highlights

• FMD vaccination induces only a temporary reduction in milk yield in high-producing Holstein cows.
• Vaccination induces only a transient increase in body temperature.
• The effect of FMD vaccination on dairy yield depends on cows’ production level.

## Graphical abstract

**Figure:**
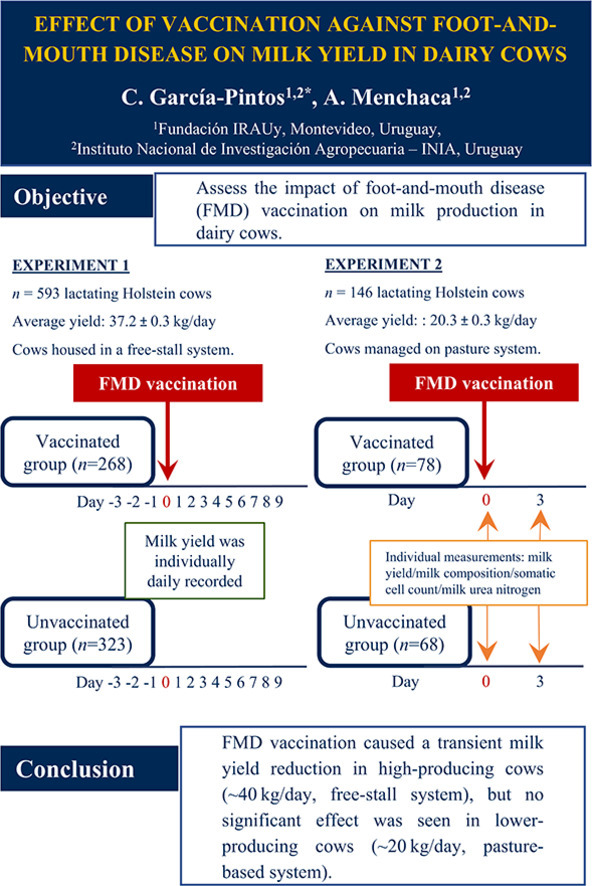

## 1 Introduction

Foot-and-mouth disease (FMD) is a highly infectious viral disease that affects cloven-hoofed ruminants (OIE, 2021). It is probably the most important livestock disease in the world in terms of economic impact, mainly because of production losses, death of animals, and restrictions on animal product trading, which act as barriers to international trade and high control/stamping out costs (James and Rushton, 2002; Knight-Jones and Rushton, 2013). The virus can be present in excretions and secretions from infected animals, including milk (Burrows et al., 1971; OIE, 2019; Sellers, 1971). Vaccination against FMD remains the most effective strategy within an integrated program aimed at preventing, controlling, and ultimately eradicating this devastating disease (Capozzo et al.*,* 2023). It has proven to be a highly useful approach in preventing dissemination and has made elimination of the disease a possibility (Barnett and Carabin, 2002; Doel, 2003; Knight-Jones et al., 2016; Parida, 2009). Herd protection reduces the opportunity for the virus to enter, replicate, and infect individuals who may be unvaccinated or lack sufficient immunity to resist (Doel, 2003).

The side effects of FMD vaccination, specifically the effects on production traits, have not been sufficiently addressed. In *Bos taurus* cows, FMD vaccination administered before 45 days of gestation has been shown to induce pregnancy failure, whereas no significant effects have been observed when vaccination occurs when gestation is more advanced (after 45 days) (García-Pintos et al., 2021). Comparable results were reported previously in *Bos indicus* cattle in Brazil (Ferrerira et al., 2016). This adverse effect in beef cows was associated with a slight increase in body temperature and local adverse reactions at the inoculation site after vaccination (García-Pintos et al., 2021), and with an increase in acute-phase protein levels and haptoglobin (Ferrerira et al., 2016; Kim et al., 2021). Although there is limited knowledge about the possible effects of FMD vaccines on milk production (Krishnaswamy et al., 2021; Yeruham et al., 2001), dairy farmers and industry often claim that FMD vaccine administration causes a temporary decline in milk yield (Athambawa et al., 2021; Krishnaswamy et al., 2021). Farmer perception of vaccine side effects may influence vaccination practices (Athambawa et al., 2021; Dernburg et al., 2007). However, scientific evidence supporting this effect remains limited and inconsistent. Therefore, further investigations are needed to provide robust evidence regarding the potential effects of FMD vaccination on milk yield in dairy cattle.

The objective of this study was to evaluate the effect of FMD vaccination on milk yield in Holstein cows at two different production levels (∼40 and ∼20 kg/day/cow) managed under distinct production systems (free-stall and pasture-based, respectively), and to assess the associated changes in body temperature following vaccination. We hypothesized that high-producing cows would be more susceptible to vaccination-related stress, resulting in a greater impact on milk yield compared with lower-producing cows.

## 2 Materials and methods

Two experiments were conducted during the period designated for FMD vaccination by the official campaign against FMD in Uruguay (*i.e*., 15 February to 15 March 2020). A total of 739 lactating female Holstein cows, privately owned by local farmers, were used in the two experiments. All cows had been previously vaccinated and revaccinated against FMD before reaching one year of age, and continued to receive annual revaccinations, with the most recent dose administered one year prior to the start of each experiment. The cows were located on two dairy farms producing an average of 37 kg/cow/day in a free-stall housing system (Experiment 1) and 20 kg/cow/day in a pasture-based system (Experiment 2). All experimental procedures were approved by the Internal Animal Care Committee of *Fundación IRAUy* (protocol number 003/2019). Animal experimentation was performed in accordance with national regulations in Uruguay (Law No. 18.611) and following the guidelines of the *Comisión Honoraria de Experimentación Animal* (CHEA), together with institutional guidelines. Within each farm, the cows were randomly assigned to two experimental groups to receive or not receive 2 mL of FMD vaccine (Bioaftogen series 945, Biogenesis Bagó, Buenos Aires, Argentina) via subcutaneous injection. According to the manufacturer, the vaccine consisted of an oil-emulsion compound containing FMD virus types O1 Campos and A24 Cruzeiro replicated in BHK suspension cell culture, inactivated with binary ethylenimine, and purified with polyethylene glycol. Vaccinations were administered subcutaneously in the neck region using a 15 mm × 18-gauge needle, which was disinfected after each use with a solution of 50% alcohol (80%) and 50% iodopovidone. The vaccine cold chain was strictly maintained, ensuring storage between 4 °C and 8 °C until administration.

### 2.1 Experiment 1

The study involved 593 lactating Holstein cows, consisting of 263 primiparous and 330 multiparous, with a body condition score (BCS) of 3.7 ± 0.1 (mean ± SEM, scale 1 to 5, from emaciated to obese, respectively; Edmonson et al., 1989). The cows had an average milk yield of 37.2 ± 0.3 kg/day/cow (ranging from 21 to 60 kg/day) and 187.8 ± 2.7 days in milk (DIM, ranging from 34 to 492 days). These lactating animals were managed under the same conditions in a free-stall housing system across three locations within the same company. The cows were milked three times a day (at 10:00 h, 18:00 h, and 02:00 h, considered the morning, afternoon, and evening milkings, respectively) and received an average of 25.5 kg of dry matter per cow from a total mixed ration, with unrestricted access to water. Only cows without clinical signs of mastitis or any other health conditions were included in the study (Ruprechter et al., 2020). On Day 0 of the experiment, the animals were randomly assigned to two experimental groups to receive (*n* = 268) or not receive (*n* = 325) the FMD vaccine. From Day −3 until Day 9, milk production was measured in terms of quantity (kg), conductivity (mS/cm), and milk flow (kg/min) (DemaTron 70 GEA Farm Technologies, Düsseldorf, Germany, endorsed by the International Committee for Animal Recording). The data were recorded (Dairy Plan C21, GEA) and analyzed via commercial software (DairyComp, Valley Agricultural Software, California, USA). Body temperature was determined from Day 0 to Day 3 postvaccination by measuring the vaginal temperature once a day (between 11:00 and 12:00) via digital thermometers (OMRON, Dalián, China) in a subset of 96 cows that received (*n* = 56) or did not receive (*n* = 40) the FMD vaccine. Vaginal temperature measurement methodology was based on previous studies in lactating dairy cows (Vickers et al., 2010; Vitali et al., 2024). All the measurements were taken by the same operator. The environmental conditions, including humidity and air temperature, were recorded to calculate the temperature‒humidity index (THI) using the following formula: THI = (0.8 × T °C) + [(RH/100) × (T °C – 14.4)] + 46.4, where T = temperature and RH = relative humidity (Mader et al., 2006). The THI scores were used as descriptive data for the environment, as follows: 81 on Day 0, 78 on Day 1, 75 on Day 2 and 72 on Day 3.

The daily milk weights from the morning, afternoon, and evening milking sessions were combined to determine the daily milk yield (kg/day). The morning milk accounted for 34.8 ± 0.1% of the daily production, the afternoon milk accounted for 31.9 ± 0.1%, and the evening milk accounted for 33.3 ± 0.1%. For data analysis, cows were classified according to daily milk production, which was calculated as the average for the three days preceding Day 0 of the experiment (37.2 ± 0.3 kg/day). For further analysis, cows were categorized into those producing above and below the average: ≥ 37.2 kg/day (mean 42.4 ± 0.3 kg/day, *n* = 276) and < 37.2 kg/day (mean 32.9 ± 0.2 kg/day, *n* = 317), respectively. Cows were also classified based on days in milk (DIM), using the herd average of 188 days as a reference: < 188 DIM (mean 111.9 ± 1.4 days, *n* = 207) and ≥ 188 DIM (mean 228.5 ± 2.0 days, *n* = 386). Cows were considered normothermic when their vaginal temperature was < 39.5 °C, and hyperthermic when it was ≥ 39.5 °C (Drillich et al., 2001).

### 2.2 Experiment 2

The study involved 146 lactating Holstein cows, 45 primiparous and 101 multiparous, with a BCS of 3.6 ± 0.1 (mean ± SEM), a milk yield of 20.3 ± 0.3 kg/cow (ranging from 9.4 to 35.9) and 260.8 ± 8.5 DIM (ranging from 14 to 584 days). The cows were managed in a pasture-based system and milked twice a day, at 05:00 h (considered morning milking) and at 16:00 h (considered afternoon milking). During the experiment, the cows grazed on a sorghum field and were supplemented twice a day with 4 kg of a balanced ration (18% vegetal protein) with unrestricted access to water. Only cows without clinical signs of mastitis or any other clinical conditions were included in the study (Ruprechter et al., 2020). The mean annual bulk milk somatic cell count (SCC) in this herd was 198.1 ± 8.7 × 1000 cells /mL, with a protein content of 3.47 ± 0.02% and a fat content of 3.53 ± 0.05%. On Day 0 of the experiment, the animals were randomly assigned to two experimental groups to receive (*n* = 78) or not receive (*n* = 68) the FMD vaccine. Milk yield was recorded on Days 0 and 3 at each milking using milk meters (J. Delgado, Scuéllamos, Ciudad Real, Spain). During the afternoon milking on these days, individual 10 mL milk samples were collected from proportional-line samplers, preserved with bronopol (2‑bromo-2-nitro-1,3-propanediol, CAS number 52-51-7), and transported to the laboratory (COLAVECO, Colonia, Uruguay) for analysis of milk components and SCC.

Cows were classified based on DIM using the herd average of 261 days as a reference: < 261 DIM (mean: 189.8. ± 2.4 days and 22.6 ± 0.4 kg/day, *n* *=* 89) and ≥ 261 DIM (mean: 375.6 ± 8.2 and 19.0 ± 0.4 kg/day, *n* *=* 57). The relationship between milk fat and protein content was calculated (fat %/protein %). Milk samples taken on Day 0 and Day 3 were analyzed for fat content (g/100 mL, %), protein content (g/100 mL, %), lactose content (g/100 mL, %) and SCC (x 1000 cells /mL). Additionally, on Day 3, milk urea nitrogen concentrations (MUN, mg /dL) were also analyzed. The SCC was performed with a somatic cell *counter* employing a *flow cytometer* based on the fluoro-opto*electronic* cell *counters* technique (Delta Instruments Combiscope FTIR 600 Dairy Analyzer, Somascope 600, North Shore, New Zealand) according to ISO 13,366–2/IDF 148–2:2006. The expanded uncertainty intralaboratory for the SCC was 0.002 log 10, and the interlaboratory uncertainty was 0.004 log 10. The milk components were analyzed via mid-infrared spectrometry (Delta Instruments Combiscope FTIR 600 Dairy Analyzer, Lactoscope 600, North Shore, New Zealand) according to ISO 9622:2013 – IDF 141. For fat content, the expanded uncertainty intralaboratory was 0.012 g/100 mL, whereas the interlaboratory uncertainty was 0.023 g/100 mL. For protein content, the expanded uncertainty intralaboratory uncertainty was 0.096 g/100 mL, and the interlaboratory uncertainty was 0.028 g/100 mL.

### 2.3 Statistical analysis

In both experiments, the mean daily milk yield was analyzed via a generalized linear mixed model (GLMM) implemented InfoStat software (Di Rienzo et al., 2020). The distribution of the variables and model residuals was evaluated prior to analysis, and data transformations were applied when necessary to meet model assumptions. The model included treatment (vaccination *vs.* no vaccination), day of the experiment, parity status (primiparous *vs*. multiparous), DIM (< 189 DIM *vs*. ≥ 189 DIM for Experiment 1, and < 261 DIM *vs.* ≥ 261 DIM for Experiment 2) and their interaction as fixed effects. In Experiment 1, the analysis was performed separately for two different milk yield levels (*i.e.*, ≥ 37.2 kg/day/cow and < 37.2). Animal identification (in both experiments, 1 and 2) and herd location (in Experiment 1) were included as random effects. In Experiment 1, body temperature was analyzed via GLMM, including the same fixed effects described above, with animal identification included as a random effect. In addition, in Experiment 1, both Pearson's and partial correlation coefficients were used to describe the relationships between variables. Specifically, the variation in milk production before vaccination (averaged over the three days preceding Day 0), average milk production on Days 1 and 2, and body temperature on Day 1 were evaluated. The logistic regression analysis was performed to estimate the intercept and slope parameters using maximum likelihood estimation for each significant continuous predictor. Probabilities were calculated as: exp(logit) / [1 + exp(logit)]. Logistic regression curves were constructed according to the coefficients provided by the interactive data analyses from InfoStat software. Polynomial regression was used to select statistical models according to the significance of the regression coefficients (*P* < 0.05) and in relation to the coefficient of determination (R2). Regression analysis was used to determine the nature of the relationship (linear, quadratic, or cubic) between each measurement, variation in milk production and body temperature before and after vaccination. In Experiment 2, the SCC and milk composition were analyzed via GLMM using InfoStat software. The SCC data were analyzed using the natural logarithm of the SCC, and the SCCs were log-transformed into a somatic cell score (SCS = log2 (SCC/100,000 cells) + 3) (Ali and Shook, 1980; Magro et al., 2023). The statistical model for MUN included the following fixed effects: treatment, DIM (< 261 *vs.* ≥ 261 DIM), parity status and their interaction. The model for milk lactose, milk fat, milk protein and the relationship between milk fat and protein included treatment, DIM, parity status, milk production level, day of the experiment and their interaction as fixed effects. Animal identification was included as a random effect.

Data from both experiments are presented as mean ± SEM. Statistical significance was set at *P* < 0.05, and tendencies were considered at 0.05 < *P* < 0.10. Non-significant results are described as not significant.

## 3 Results

### 3.1 Experiment 1

**Fig. 1:**
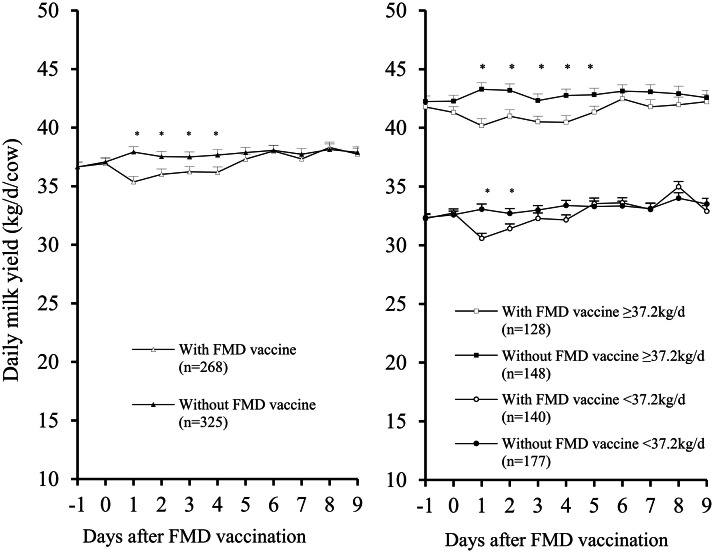
Daily milk yield (mean ± SEM) of Holstein cows that received or did not receive a dose of foot-and-mouth disease (FMD) vaccine on Day 0. Data are expressed as the main effect of treatment (left panel) and after evaluation regarding the milk yield level (right panel). Asterisks (*) represent significant differences (*P* < 0.05)*.*

The daily milk yield was affected by the administration of the FMD vaccine, by the day of the experiment and by their interaction (*P* *<* 0.05). The data were compared separately to milk yield levels in high- and medium-producing cows. In high-producing cows (≥ 37.2 kg/day), milk yield from Day 1 to Day 5 was lower in vaccinated than unvaccinated cows (40.7 ± 0.3 kg/day *vs.* 42.9 ± 0.3 kg/day, respectively) (*P* < 0.05). In medium-producing cows (< 37.2 kg/day/cow), milk yield was lower on Days 1 and 2 in vaccinated than unvaccinated cows (31.0 ± 0.3 kg/day *vs.* 32.9 ± 0.3 kg/day, respectively) (*P* < 0.05). The results are shown in Fig. 1.

During the evaluated period, milk yield varied significantly based on DIM, with cows ≤ 188 DIM producing 42.8 ± 0.2 kg/day compared with 34.3 ± 0.1 kg/day for those ≥ 189 DIM (*P* < 0.05). Parity status also influenced milk yield, with primiparous cows producing 35.4 ± 0.1 kg/day milk and multiparous cows producing 38.8 ± 0.1 kg/day milk (*P* < 0.05). No significant interactions were detected between treatment (vaccination) and either DIM or parity status. The detailed results can be found in Table 1.

**Table 1:** Daily milk yield (kg/day/cow; mean and common SEM) in lactating Holstein cows following foot-and-mouth disease (FMD) vaccination (Day 0) compared to an unvaccinated control group.

		Day 0	Day 1	Day 2	Day 3	Day 4	Day 5	SEM
Main effects								
FMD vaccine	With (*n* = 268)	36.9^a^	35.4^b^	36.0^b^	36.3^b^	36.2^b^	37.3^a^	0.4
	Without (*n* = 325)	37.1^a^	37.9^a^	37.5^a^	37.5^a^	37.7^a^	37.9^a^	0.4
DIM	≤ 188 days (*n* = 207)	41.8^a^	41.3^a^	43.0^a^	42.0^a^	42.4^a^	42.7^a^	0.5
	≥ 189 days (*n* = 386)	34.6^b^	33.3^b^	33.8^b^	34.1^b^	34.1^b^	34.8^b^	0.4
Parity	Primiparous (*n* = 263)	35.4^a^	34.3^b^	34.7^b^	35.1^b^	35.1^b^	35.7^b^	0.4
	Multiparous (*n* = 330)	38.4^b^	38.5^a^	38.5^a^	38.3^a^	38.5^a^	39.0^a^	0.5

Different letters for the same column for each main effect indicate *P* *<* 0.05. No interaction between FMD vaccine treatment and days in milk (DIM) or between FMD vaccine treatment and parity status was detected.

**Fig. 2:**
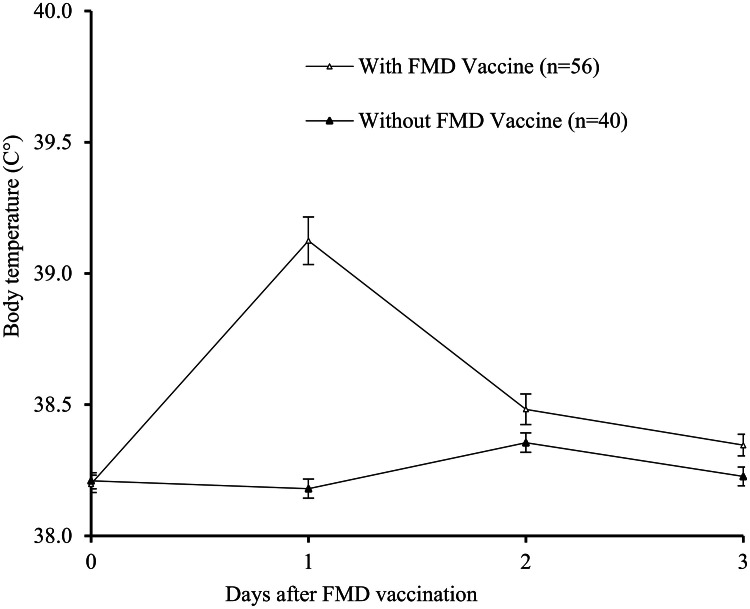
Body temperature (mean ± SEM) of dairy Holstein cows that received or did not receive a dose of FMD vaccine on Day 0. A significant difference was found on Day 1 (*P* < 0.05*).*

**Fig. 3:**
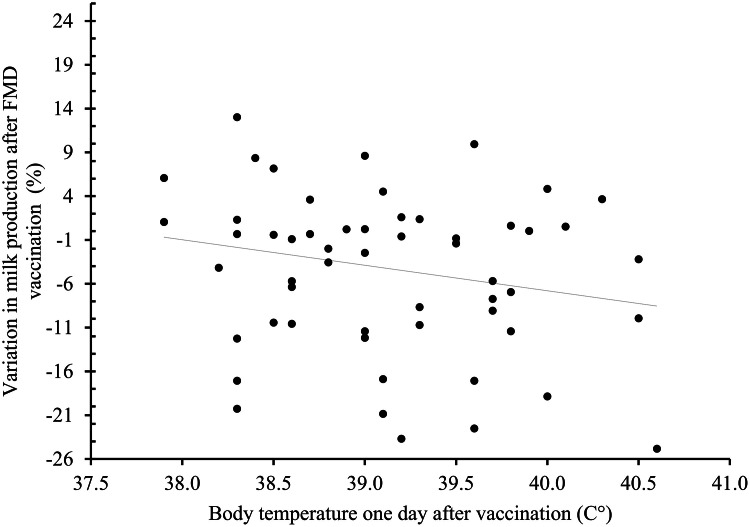
Association between variation in milk production before and after vaccination (average milk production on Days 1 and 2 was calculated) and body temperature on Day 1 in dairy Holstein cows that received a dose of FMD vaccine on Day 0 (*n* = 56). Correlation between the milk yield and body temperature after vaccination was not statistically significant (*R* = 0.04).

Body temperature depended on treatment, day, and their interaction (*P* < 0.05). On Day 1, the temperature was greater in vaccinated than unvaccinated cows (39.1 ± 0.1 *vs*. 38.2 ± 0.1 °C, respectively; *P* *<* 0.05). The temperature of the vaccinated animals declined on Day 2 to values comparable to those of unvaccinated cows (38.5 ± 0.1 °C *vs*. 38.4 ± 0.1 °C, respectively; *P* > 0.05). The results are presented in Fig. 2. While 34% of the vaccinated group experienced a body temperature higher than 39.5 °C (hyperthermia), none of the cows in the unvaccinated group experienced hyperthermia during the experiment (*P* < 0.05). The mean values are shown in Table 2. Milk production, DIM, and parity had no significant influence on temperature variation, and no significant interaction was detected between these factors and FMD vaccine treatment. No correlation was found between daily milk production and body temperature in either vaccinated or unvaccinated cows. The relationships between variations in milk production before and after vaccination and body temperature on Day 1 for vaccinated cows are shown in Fig. 3.

**Table 2:** Percentage of dairy Holstein cows suffering hyperthermia (≥ 39.5 °C) after foot-and-mouth disease (FMD) vaccination (Day 0) compared with unvaccinated cows.

	Day 0	Day 1	Day 2	Day 3
With FMD vaccine (*n* = 56)	0% (0/56)	34% (19/56)	2% (1/56)	2% (1/56)
Without FMD vaccine (*n* = 40)	0% (0/40)	0% (0/40)	0% (0/40)	0% (0/40)
*P* value	-	< 0.01	0.40	0.40

### 3.2 Experiment 2

**Fig. 4:**
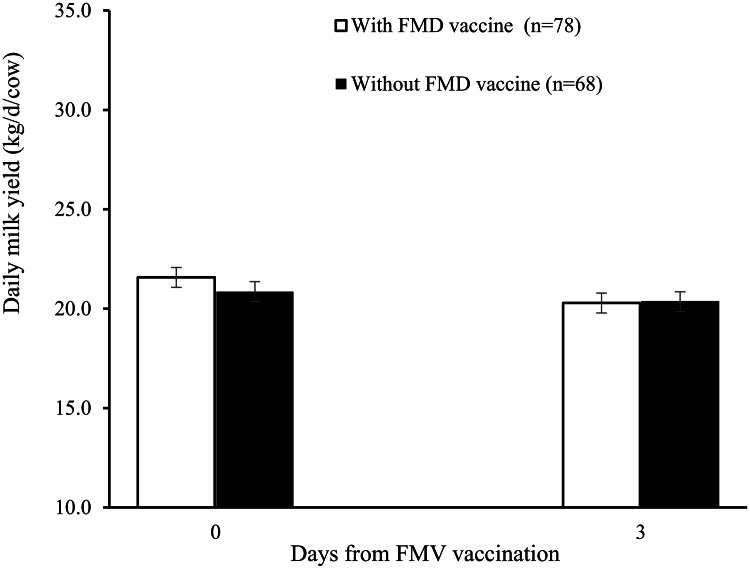
Daily milk yield (mean ± SEM) of Holstein cows that received or did not receive a dose of FMD vaccine on Day 0. Treatment, day, or the interaction treatment*day had no significant effect.

FMD vaccination had no effect on milk yield, which was 20.3 ± 0.4 and 20.4 ± 0.4 kg/day/cow for vaccinated and unvaccinated cows, respectively. The results are presented in Fig. 4. Milk content and SCC were not influenced by FMD vaccine administration. The concentrations of MUN tended to be lower in vaccinated than unvaccinated cows (21.3 ± 0.2 *vs.* 22.3 ± 0.3, respectively) (*P* = 0.06). On Day 3, the milk protein content tended to be lower in vaccinated than unvaccinated cows (3.48 ± 0.05 *vs*. 3.66 ± 0.05, respectively; *P* = 0.08). Cows producing < 20.3 kg/day had greater protein content than those producing ≥ 20.3 kg/day on Day 0, 3.72 ± 0.03 *vs.* 3.44 ± 0.03%, respectively (*P* *<* *0.05*). Milk protein content, SCC and milk yield were influenced by DIM (*P* *<* *0.05*). The milk protein content and SCC were greater in cows with ≥ 261 days than in those with < 261 DIM (3.77 ± 0.04 *vs*. 3.47 ± 0.03% of protein), and the SCC was 230 ± 19 *vs.* 136 ± 12 × 1000 cs /mL, respectively (*P* *<* *0.05*). Moreover, on Day 3, the milk yield of cows with ≥ 261 DIM was 18.1 ± 0.3 kg/cow, whereas that of cows with < 261 DIM was 21.8 ± 0.3 kg/cow (*P* < 0.05). The statistical interactions are presented in Table 3, and the results for different experimental groups are detailed in Table 4.

**Table 3:** Main effects and statistical interactions (*P* values) of FMD vaccination (Day 0) on milk composition and SCS of lactating Holstein cows (*n* = 146). Results correspond to measurements obtained on Day 3.

		Milk yield	Fat	Protein	Protein/fat	Lactose	SCS	MUN
Main effects								
FMD vaccine	With *vs.* Without	0.45	0.87	0.08	0.42	0.18	0.22	0.06
Day	Day 0 *vs.* Day 3	0.08	0.34	0.08	0.73	0.08	0.61	-
Parity	Primiparous *vs.* multiparous	0.35	0.89	0.18	0.70	0.11	0.23	0.91
Milk yield level	kg/day Higher (≥20.3 kg/day) *vs*. Lower (<20.3 kg/day)	-	0.79	<0.01	0.86	0.41	0.43	0.92
Days in milk	< 261 d *vs.* ≥ 261d	0.01	0.43	0.05	0.95	0.70	0.02	0.80
FMD interactions								
FMD vaccine x Day		0.39	0.82	0.09	0.87	0.10	0.83	-
FMD vaccine x parity status		0.66	0.25	0.30	0.76	0.19	0.31	0.25
FMD vaccine x milk production		0.47	0.11	0.31	0.28	0.56	0.99	0.22
FMD vaccine x DIM		0.08	0.99	0.11	0.75	0.47	0.39	0.92

DIM = days in milk; MUN = milk urea nitrogen concentrations.

SCS = log2 (somatic cell count/100,000 cells) + 3.

**Table 4:** Milk composition and somatic cell count (SCC) of lactating Holstein cows after administration of the FMD vaccine (n = 78) compared with unvaccinated cows (n = 68).

Milk component	Day of the experiment	With FMD vaccine	Without FMD vaccine	SEM
Fat (%)	Day 0	3.77	3.69	0.10
	Day 3	3.89	3.95	0.10
Protein (%)	Day 0	3.61	3.63	0.04
	Day 3	3.48	3.66	0.04
Protein/fat	Day 0	1.01	1.01	0.02
	Day 3	0.95	0.96	0.02
Lactose (%)	Day 0	4.80	4.85	0.01
	Day 3	4.72	4.73	0.02
SCC (1000 cells /mL)	Day 0	187	195	24
	Day 3	159	148	18

Values are presented as mean ± SEM. No significant differences were observed between treatments.

## 4 Discussion

The main finding of this study was that, in high-producing Holstein cows (around 40 kg/day per cow), FMD vaccination was associated with a slight decrease in milk yield during the five days following vaccination, an effect that was not evident in cows producing approximately 20 kg/day. The vaccine also induced an increase in body temperature on the day after vaccination.

The milk yield in cows with a daily production ≥ 37.2 kg decreased by an average of 5.0% during the five days following the administration of the FMD vaccine. Vaccination induces an immune response involving activation of the innate immune system and the release of pro-inflammatory cytokines. This process is associated with systemic effects such as increased body temperature, reduced feed intake, and lethargy, which may contribute to a temporary decrease in milk production. In addition, the activation of the immune system leads to a redistribution of nutrients and energy toward immune function rather than milk synthesis, further explaining the observed reduction in milk yield (Lippolis et al., 2017). Despite limited information on the effect of the FMD vaccine on dairy production, the available evidence is mainly based on case reports or observational studies under specific field conditions. For example, a case report published in Israel (Yeruham et al., 2001) described a 21.5% decrease in daily milk production for seven days following subcutaneous administration of an FMD vaccine containing aluminum hydroxide gel and saponin as adjuvants in a herd with an average production of approximately 30 kg/day/cow. This reduction was greater than that observed in the present study, likely reflecting differences in vaccine formulation and adjuvants. In particular, the vaccine used in the present study contained an oil-based adjuvant and did not include saponin, which may partly explain the milder effects observed. In contrast, a study conducted in India (Krishnaswamy et al., 2021), using a trivalent oil-based inactivated vaccine administered intramuscularly (2 mL), reported no significant differences in milk yield between vaccinated and unvaccinated cows, including both Bos indicus (Deoni cattle) and crossbred Holstein × Bos indicus animals. Overall, current evidence on the effect of FMD vaccination on milk production remains limited and inconsistent. In this context, the present study provides a structured and comparative evaluation of the effect of vaccination on milk yield under field conditions, offering novel and robust evidence in this area. Regarding milk composition, SCC, and MUN, no consistent or biologically relevant changes were observed following vaccination, suggesting that the main effect of vaccination was on milk yield rather than milk quality parameters.

The administration of the FMD vaccine induced hyperthermia in 34% of lactating cows on the day following vaccination. Similarly, a previous study reported that 28% of animals presented fever within 1–2 days post-vaccination (García-Pintos et al., 2021). However, lactating dairy cows differ from beef cattle in their metabolic status due to the high energy demands of milk production, which may influence the physiological response to vaccination. Within this context, the present study provides specific evidence on the occurrence and magnitude of post-vaccination hyperthermia in dairy cows under field conditions. In another report conducted on indicus and crossbred cows using a vaccine from a different manufacturer, FMD vaccination resulted in a significant increase in body temperature, ranging from 0.4 °C to 0.6 °C (Raina et al., 2023). Although the precise mechanisms linking hyperthermia to changes in milk production are not fully understood, several physiological pathways have been proposed. These mechanisms involve the activation of numerous dendritic cells, enhancing interactions between antigen-presenting cells and helper T lymphocytes in the lymph nodes (Baccili et al., 2019). The release of cytokines into the circulatory system induces inflammation (Burny et al., 2017). These cytokines then reach the hypothalamus and other centers in the central nervous system, triggering the production of prostaglandin-E2, which in turn induces hyperthermia, lethargy, and reduced appetite (Bosch et al., 1997; Kuhla, 2020). Reduced feed intake decreases nutrient availability, potentially leading to a decline in milk production (Kadzere et al., 2002; Polsky et al., 2017). Nevertheless, in our study, no clear association was observed between hyperthermia and changes in milk yield, as milk production decreased in both hyperthermic and normothermic cows following vaccination. Hyperthermic cows are unable to engage the normal glucose-sparing mechanisms used by normothermic animals to maximize milk yield during periods of nutrient insufficiency (Wheelock et al., 2010). While hyperthermia may contribute to the reduction in milk yield following FMD vaccination, it is likely not the sole factor involved. Inflammatory responses and decreased feed intake may also play significant roles. Further research is required to better elucidate the specific mechanisms by which FMD vaccination affects milk production in dairy cows.

The outcomes observed across both experimental herds, which included cows producing between 10 and 60 kg/day of milk, suggest that the negative impact of FMD vaccination on milk yield primarily affects high-producing cows. Milk production decreased for five days in cows producing 42 kg/day, whereas the reduction lasted only two days in those producing 32 kg/d. Conversely, vaccination did not affect milk yield, milk composition, or SCC in cows producing 20 kg/d. Other factors, such as differences in nutrition, management systems, and experimental conditions between herds, should be considered when interpreting these results, as they may have contributed to the variability observed between the two experiments. Although this study did not investigate the underlying mechanism for these differences between high- and low-producing dairy cows, it is possible that the metabolic demands associated with increased milk production play a role. Greater energy requirements for milk production are often linked to metabolic and immunological disruptions (Baccili et al., 2019), and reduced feed intake due to discomfort or loss of appetite after vaccination (Rodrigues et al., 2015) may disproportionately affect milk yield in high-production cows compared with that in moderate- or low-production cows. In this context, evidence suggests that FMD vaccination can lead to a reduction in dry matter intake of approximately 0.7–0.8 kg (Raina et al., 2023), which may partially account for the decline in milk yield observed in this study.

Vaccination is essential for controlling and eradicating FMD, especially in countries like Uruguay, where the last outbreak in 2001 led to significant losses, including a 1.9% reduction in Gross Domestic Product between 2001 and 2003 and amounting to approximately $730 million (Iriarte et al., 2023; Knight-Jones and Rushton, 2013). It has been reported that this disease reduces milk yield by 50% to 80% (Barasa et al., 2008; Bayissa et al., 2011; Jemberu et al., 2014; Onono et al., 2013), increases the likelihood of mastitis and abortions, and delays the interval to conception (James and Rushton, 2002). Maintaining adequate vaccination coverage is essential, as low coverage has historically allowed FMD to reach epidemic proportions (Fernando, 1969). In terms of indirect costs, vaccination is widely recognized as being more cost-effective than any control strategy that does not involve vaccination (Barratt et al., 2019). Our study revealed a mild, short-term decrease in milk yield lasting only five days in high-producing cows following FMD vaccination, with no effect observed in lower-producing cows. However, these losses are minimal compared to the devastating impact an FMD outbreak can have on the livestock industry, as well as on the economy of a country or entire region. Our findings provide robust evidence on the effect of FMD vaccination on milk yield, demonstrating only slight effects in high-producing cows and challenging common misconceptions, unverified reports, and anecdotal beliefs regarding this issue.

## 5 Conclusion

FMD vaccination caused a transient reduction in milk yield only in high-producing Holstein cows under a free-stall management system, with no significant effects in lower-producing cows managed on a pasture-based system. Vaccination was also associated with a transient increase in body temperature. These findings suggest that high-producing cows may be more susceptible to vaccination-related stress. Overall, the impact of FMD vaccination on dairy production is minimal when considering its crucial role in controlling this devastating disease.

## Funding

This work was supported by the Instituto Nacional de Investigación Agropecuaria of Uruguay.

## Statement for studies in animals

All experimental procedures involving animals were approved by the Internal Animal Care Committee of Fundación IRAUy (protocol number 003/2019) and were conducted in accordance with national regulations governing animal experimentation in Uruguay (Law No. 18.611) and the guidelines of the Comisión Honoraria de Experimentación Animal (CHEA).

## CRediT authorship contribution statement

**C. García-Pintos:** Writing – original draft, Investigation, Formal analysis, Conceptualization. **A. Menchaca:** Writing – review & editing, Supervision, Project administration.

## Declaration of competing interest

C. García-Pintos reports financial support from the National Agricultural Research Institute of Uruguay and administrative support from Fundación IRAUy. All other authors declare no competing financial interests or personal relationships that could have influenced the work reported in this paper.
